# COVID-19 outbreak among French firefighters, Marseille, France, 2020

**DOI:** 10.2807/1560-7917.ES.2021.26.41.2001676

**Published:** 2021-10-14

**Authors:** Guillaume André Durand, Franck de Laval, Albane de Bonet d’Oléon, François Xavier Le Flem, Yann Morin, Cyril Badaut, Gilda Grard, Constance Brossier, Marion Fossier, Aissata Dia, Flavie Letois, Manon Geulen, Géraldine Piorkowski, Jean-Baptiste Meynard, Frank Peduzzi, Isabelle Leparc-Goffart, Vincent Pommier de Santi

**Affiliations:** 1 French Armed Forces Biomedical Research Institute, National Reference Laboratory for Arboviruses, Marseille, France; 2 Unité des Virus Émergents (UVE: Aix-Marseille Univ-IRD 190-Inserm 1207), Marseille, France; 3 French Military Health Service, French Armed Forces Centre for Epidemiology and Public Health (CESPA), Marseille, France; 4 Aix-Marseille University, INSERM, IRD, SESSTIM (Economic and Social Sciences, Health Systems, and Medical Informatics), Marseille, France; 5 Marseille Battalion of Navy Firefighters, 9 boulevard de Strasbourg, 13233 Marseille Cedex 20, France; 6 Microbiology and Infectious Diseases Department, Institut de Recherche Biomédicale des Armées (IRBA), Brétigny-sur-Orge Cedex, France; 7 University Hospital Institute Méditerranée Infection, Marseille, France; 8 Aix-Marseille University, IRD, AP-HM, SSA, VITROME, Marseille, France

**Keywords:** COVID-19, SARS-CoV-2, presymptomatic, asymptomatic, neutralisation assay, serology

## Abstract

We investigated a COVID-19 outbreak at a fire station in Marseille, France. Confirmed cases were defined as individuals with positive SARS-CoV-2 reverse transcription (RT)-PCR and/or neutralising antibodies. All 85 firefighters at work during the outbreak period were included after questioning and sampled for RT-PCR and viral neutralisation assay. Twenty-three firefighters were confirmed positive, 19 of them were symptomatic, and four asymptomatic cases were confirmed by virus neutralisation. A total of 22 firefighters had specific neutralising antibodies against SARS-CoV-2. Neutralising antibodies were found in four asymptomatic and 18 symptomatic cases. Eleven symptomatic cases had high titres (≥ 1:80). The earliest detection of neutralising antibodies was 7 days after symptom onset, and 80% had neutralising antibodies 15 days after onset. One viral culture was positive 13 days after onset. The attack rate was 27%. We identified two introductions of the virus in this outbreak, through a presymptomatic and a paucisymptomatic case. Asymptomatic cases were not the source of a third generation of cases, although they worked without wearing a mask, indicating that asymptomatic cases did not play a significant role in this outbreak. Management and strategy based on early research of clinical signs associated with self-quarantine was effective.

## Background

Complete investigations of coronavirus disease (COVID-19) outbreaks in close-contact communities such as fire stations are scarce. Such a context offers the opportunity to track virus transmission and increase knowledge of COVID-19 epidemiology. Before the emergence of variants in 2020, the basic reproduction number (R_0_) of severe acute respiratory syndrome coronavirus 2 (SARS-CoV2) Wuhan strain was estimated at between 2 and 3.5 [1], and the commonly accepted median incubation time was 5.1 days [2]. It was suspected that presymptomatic (i.e. detection of the virus before symptom onset) as well as asymptomatic (i.e. detection of the virus without any symptoms) individuals were able to transmit the virus and can therefore play a role in the transmission chain [3,4]. General preventive measures such as social distancing, hand hygiene and face masks reduce R_0_ by two thirds and could prevent transmission by presymptomatic, asymptomatic and symptomatic cases [5]. After infection, cellular and humoral immune responses are induced [6,7]. The latter can easily be measured with serological assays, and neutralising antibodies appear in the 2 weeks following infection. However, at the beginning of the COVID-19 pandemic, little was known about their kinetics, their duration, the effective clinical protection they afford and their link with COVID-19 severity [8]. Until more data are available, it is commonly accepted that the presence of neutralising antibodies leads to clinical protection from reinfection with the same strain [9,10].

By 13 March 2020, 27,000 new COVID-19 cases had been reported in European countries, mainly in Italy, Spain and France (2,876 cases) [11]. Considering the strong increase in COVID-19 incidence, the French population was placed under lockdown on 17 March. The first case in the city of Marseille was reported on 3 March, and a cumulative total of 93 cases was reached for the entire department of Bouches-du-Rhônes (2 million inhabitants) on 13 March. French firefighters are at high risk of infection because they live in close quarters in crowded barracks while on duty and can be exposed during rescue operations. A cluster at a fire station, given the close contact, could represent an opportunity to highlight epidemiological and transmission factors. Here we investigate a COVID-19 outbreak at a fire station in Marseille.

### Outbreak detection

Fire services in France are organised locally and cover the entire country. In the cities of Paris and Marseille, firefighters belong to the military, whereas in other cities they are civilians. In Marseille, they belong to the Navy Fire Battalion. A particularity of French firefighters is that they also provide emergency medical assistance. Therefore, they use both fire trucks and, as paramedics, ambulance vehicles to transport injured people and patients to the hospital, including those with COVID-19. During 24-h on-call shifts, firefighters eat meals together and share accommodations, sleeping in rooms with between two and four beds, and therefore live in close contact with one another. The outbreak described here emerged at one of the 22 fire stations in Marseille. All 91 firefighters of this fire station were men aged between 20 and 51 years (average: 29 years). They benefit from close medical supervision at a medical facility located at the station. At the onset of COVID-19 outbreak in France, the battalion’s medical support service decided to perform systematic quantitative reverse transcription PCR (RT-PCR) on a nasopharyngeal swab for all symptomatic individuals (diagnosis stage). All symptomatic men were isolated at their home until resolution of their symptoms, regardless of whether their RT-PCR result was positive or negative, according to French regulation at that time [12]. The definition of the symptoms was broad, including mild symptoms such as a cough and runny nose. The RT-PCR test was performed at a diagnostic facility before this investigation started. 

The outbreak began on 16 March 2020 with two symptomatic individuals later confirmed as SARS-CoV-2 infected. At the end, it included a total of 19 symptomatic men, of whom 13 were found positive by RT-PCR. 

Despite this, the fire station continued to operate during the outbreak. We describe the outbreak investigation, the chain of SARS-CoV-2 transmission, and the workplace safety plan implemented at the fire station to contain the spread of the virus.

## Methods

### Study design

The epidemiological investigation began on 2 April, 17 days after symptom onset of the index cases (16 March). Active case detection among service members of the fire station was performed in order to limit the spread of the outbreak. Given the close contact between firefighters, all were considered as potentially exposed to SARS-CoV-2 during the outbreak. To identify all cases, we performed a survey and sampling for all firefighters present during the outbreak. Firstly, this strategy allowed us to confirm all cases, including asymptomatic cases and symptomatic firefighters not already confirmed by RT-PCR, in order to explain the transmission chain. Secondly, it allowed us to determine if the previously confirmed cases were still positive at the investigation stage, i.e. several days after the first RT-PCR test. All 91 firefighters of the fire station were offered the possibility to provide a nasopharyngeal sample for a SARS-CoV-2 RT-PCR test and a blood sample for a virus neutralisation assay.

### Laboratory investigation

#### SARS-CoV-2 RT-PCR

We collected the results of RT-PCR tests performed before this investigation (during the diagnosis stage). Starting at the investigation stage, detection of the viral genome in a nasopharyngeal sample was performed using a previously published RT-PCR system [13]. After automated RNA extraction (QiaSymphony, Qiagen, Hilden, Germany) of 200 µL of sample, the E gene of SARS-CoV-2 was amplified by RT-PCR. The extraction process was controlled using MS2 phage and the amplification process using synthetic E gene RNA. A test was considered positive at a quantification cycle threshold (Cq) ≤ 35.

#### Virus neutralisation assay

Detection of neutralising antibodies was performed using virus neutralisation assay for the identification of cases who were PCR-negative because they had already recovered, and for the identification of asymptomatic people. This technique was previously found to be the most specific for SARS-CoV-2 antibody detection. Sera were filtered using a 0.22 µm filter, then underwent twofold serial dilution from 1:10 to 1:80 in a 96-well plate. We added 100 µL of diluted serum to 100 µL of virus suspension containing a median tissue culture infectious dose of 100 TCID_50_ of human SARS-CoV-2 (strain BavPat1/2020, European Virus Archive GLOBAL), incubated it for 1 h at 37 °C with 5% CO_2_, and added it to Vero cells (ATCC CCL-81, 1.3 × 10^5^ cells/well). After 4 days of incubation, we investigated the cytopathic effect (CPE) by light microscope. The serum neutralising titre was calculated as the inverse of the highest dilution resulting in 50% reduction of infectivity. We defined high titres when the highest dilution was ≥ 80.

#### Viral culture

All samples with positive RT-PCR results were inoculated in Vero cells. In brief, 200 µL of sample was incubated in Vero cells (ATCC CCL-81) previously grown to subconfluence. After 1 h of incubation at 37 °C with 5% CO_2_, 3 mL of Dulbecco's Modified Eagle Medium (DMEM) with 2% fetal bovine serum was added followed by 6 days of incubation before one systematic subculturing was performed. The CPE were investigated by a trained operator, and RT-PCR of the supernatant was performed for all samples. All experiments except RT-PCR were performed in a BSL-3 facility.

### Epidemiological investigation

#### Case definition

Cases were defined according to laboratory results: confirmed SARS-CoV-2-infected cases were those with positive SARS-CoV-2 RT-PCR results and/or neutralising antibodies. Among confirmed cases, we differentiated symptomatic and asymptomatic cases. All others were considered as SARS-CoV-2-exposed cases. An asymptomatic infection was defined by the presence of neutralising antibodies or a positive RT-PCR test without a history of symptoms.

#### Data collection

All investigated firefighters were questioned by an epidemiologist in a face-to-face interview. We collected information about symptom history, clinical signs, previous laboratory analyses performed and their results, and exposure to someone with COVID-19 in their family and social circle. We also collected their work schedule for the preceding weeks in order to trace contacts within the fire station.

### Ethical statement

All participants received information about the investigation and the disease, were volunteers to participate and gave their consent, also for the publication of the investigation. According to French regulation, as this was an outbreak with immediate public health threat, ethical approval was not required for this investigation.

## Results

### Laboratory investigation

#### SARS-CoV-2 RT-PCR

**Figure 1:**
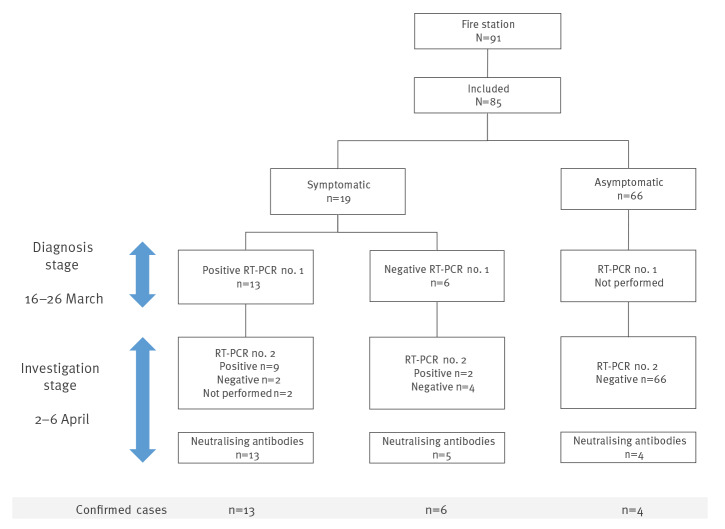
Flowchart of cases at time of diagnosis and time of investigation and results of RT-PCR and neutralisation assays, COVID-19 outbreak at a fire station, Marseille, France, 2020 (n = 85)

Eighty-five of the station’s 91 firefighters worked during the outbreak and were included in the investigation. They were sampled for detection of the SARS-CoV-2 genome in a nasopharyngeal swab (n = 83) and/or neutralising antibodies in serum (n = 85) (Figure 1). Among them, 19 were identified as symptomatic and 13 of them had already been confirmed by RT-PCR at the diagnosis stage (Table 1, Figure 1). Nine of the 13 were still RT-PCR-positive at the investigation stage, with an average Cq of 30.6 cycles (range: 20–34). The six other symptomatic individuals were confirmed at the time of the investigation: one with a positive RT-PCR test (without neutralising antibodies), one with both a positive RT-PCR test and neutralising antibodies, and four only by virus neutralisation. For the two who were RT-PCR-negative at the diagnosis stage and RT-PCR-positive (Cq 29 and 36) at the investigation stage, the time between symptom onset and the investigation was respectively 9 and 18 days. The case with a Cq of 36 (above the defined threshold) was considered positive given a typical RT-PCR amplification curve. We classified all 19 symptomatic cases as confirmed, 15 confirmed by RT-PCR and four only by virus neutralisation assay (Table 1, Figure 1). All the RT-PCR tests were negative for the 66 asymptomatic participants, but four of them displayed specific SARS-CoV-2 neutralising antibodies and were classified as confirmed cases (Table 1, Figure 1). In all, 23 firefighters were confirmed as infected with SARS-CoV-2.

SARS-CoV-2: severe acute respiratory syndrome coronavirus 2.

**Table 1:** Laboratory diagnosis of confirmed SARS-CoV-2-infected cases according to time between symptom onset and sampling, COVID-19 outbreak at a fire station, Marseille, France, 2020 (n = 23)

Case number	Symptom duration (days)	Duration 1^a^ (days)	RT-PCR 1 n = 18	Duration 2^b^ (days)	RT-PCR 2 n = 21	Neutralising antibody titre
**Symptomatic**						
1	> 13	3	+	13	+	1:80
2	7	1	+	14	+	1:20
3	> 16	3	+	16	+	1:40
4	> 13	0	+	13	+	1:40
5	> 12	1	+	12	+	1:80
6	11	4	+	16	+	1:80
7	8	2	+	9	+	1:40
8	> 13	2	+	9	+	1:80
9	> 15	4	+	15	+	1:80
10	12	2	+	15	−	1:80
11	> 9	1	+	9	−	1:80
12	4	1	+	15	NA^c^	1:40
13	> 17	5	+	32	NA^d^	1:80^d^
14	9	1	−	18	+	Negative^e^
15	5	2	−	9	+	1:40
16	12	11	−	17	−	1:160
17	5	1	−	7	−	1:80
18	> 13	6	−	13	−	1:40
19^f^	1	NA	NA	16	−	1:160
**Asymptomatic**						
20	NA	NA	NA	NA	−	1:40
21	NA	NA	NA	NA	−	1:80
22	NA	NA	NA	NA	−	1:80
23	NA	NA	NA	NA	−	1:40
**Total positive**		**13**		**11**		**22**

+: positive; −: negative; COVID-19: coronavirus disease; NA: Not applicable; SARS-CoV-2: severe acute respiratory syndrome coronavirus 2.

^a^ Duration 1 is the time between symptom onset and the first RT-PCR test performed.

^b^ Duration 2 is the time between symptom onset and the investigation (RT-PCR and neutralising antibody test).

^c^ This case did not provide a nasal sample.

^d^ This case was absent on the day of the investigation but a blood sample was taken 32 days later.

^e^ This case was negative in two assays performed 18 and 32 days after symptom onset.

^f^ This case was not initially identified as a possible case and therefore not initially tested.

#### Virus neutralisation assay

Twenty-two firefighters (26%) had anti-SARS-CoV-2 neutralising antibodies (Table 1). Neutralising antibodies were found in 18 of the 19 symptomatic cases, with a high titre (≥ 1:80) for 11 of them. The one case who was negative for neutralising antibodies was tested twice for that, 18 and 34 days after symptom onset, despite an initial positive RT-PCR test. High titres were found in two of the four asymptomatic cases. The geometric mean titre (GMT) was 63.5 and 50.4, respectively, for symptomatic and asymptomatic cases. The earliest appearance of neutralising antibodies was 7 days after symptom onset, and 14 of 18 had neutralising antibodies 15 days after symptom onset.

#### Viral culture

We performed a viral culture on the 11 RT-PCR-positive samples at the investigation stage. A CPE was observed for one sample taken 13 days after onset of symptoms and with an Cq value of 20. This case initially had headaches without fever, fatigue, myalgia, rhinitis, anosmia and dysgeusia. This suggests that this patient was still contagious at the time of the investigation, since he still had symptoms, i.e. anosmia and dysgeusia. The viral strain was sequenced (GenBank accession number: MT787505) and displayed the spike D614G mutation, previously related to increased infectivity [16].

### Epidemiological investigation

**Figure 2:**
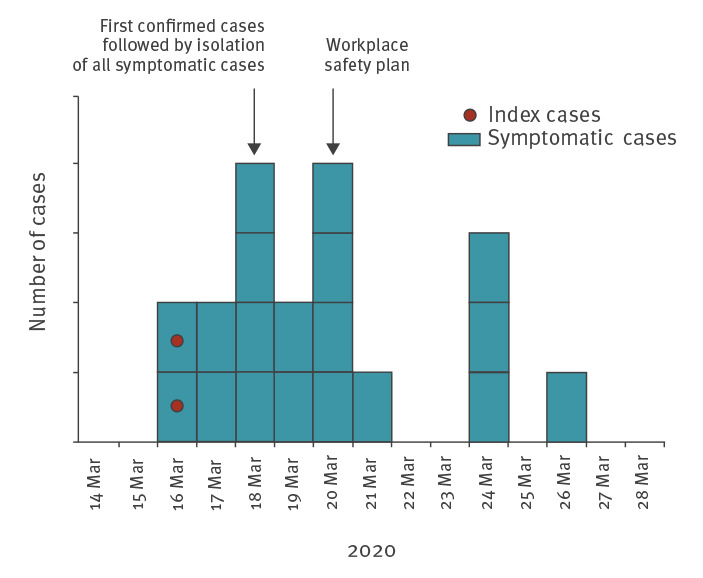
Epidemic curve of symptomatic confirmed cases, COVID-19 outbreak at a fire station, Marseille, France, 2020 (n = 19)

The final attack rate of this outbreak was 27% (23/85). The outbreak was noticed through two distinct index cases, Cases 13 and 16 (Figure 2). They declared symptoms on 16 March and one of them was present at the fire station on that day. Sequencing of the virus was not possible for them because of negative RT-PCR at investigation time and negative virus isolation (Table 1). Since they did not work together on the same team during the preceding week, we consider them as two separate introductions of the virus into the fire station. The first case (Case 13) initially developed a febrile cough with diarrhoea and asthenia on 16 March, 6 days after meeting his sibling who presented a cough and asthenia without laboratory diagnosis. The origin of contamination of the second case (Case 16) was unknown. 

COVID-19: coronavirus disease.

**Figure 3:**
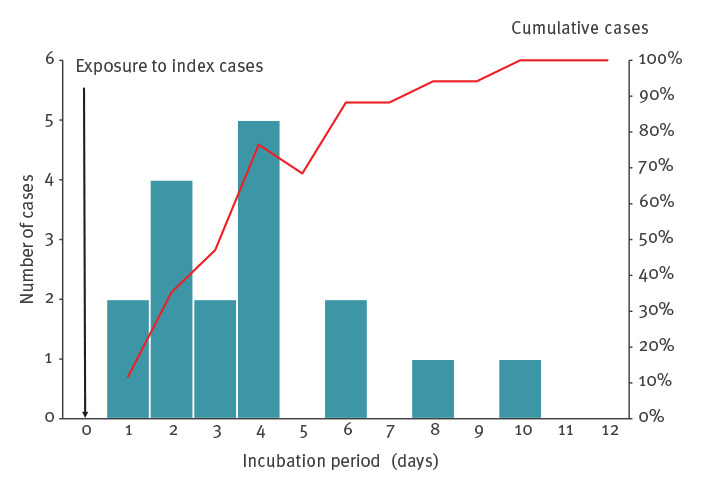
Distribution of confirmed COVID-19 cases and cumulative proportion (red line) by incubation time, COVID-19 outbreak at a fire station, Marseille, France, 2020 (n = 17)

Among the 19 symptomatic confirmed COVID-19 cases, nine were still sick at the time of the investigation. The most common symptoms were headaches (14 cases), myalgia (14 cases), asthenia (13 cases), anosmia (13 cases), dysgeusia (12 cases) and fever (nine cases). All had mild symptoms and none required hospitalisation. One case had only asthenia for 48 h. For the 10 who had already recovered when the investigation started, the average duration of symptoms was 8 days (range: 1–13). Two patients had only headaches, anosmia, dysgeusia and rhinitis. The median incubation time, calculated considering the first contact at work with one of the two index cases, was 4 days (range: 1–10) (Figure 3). 

COVID-19: coronavirus disease.

**Figure 4:**
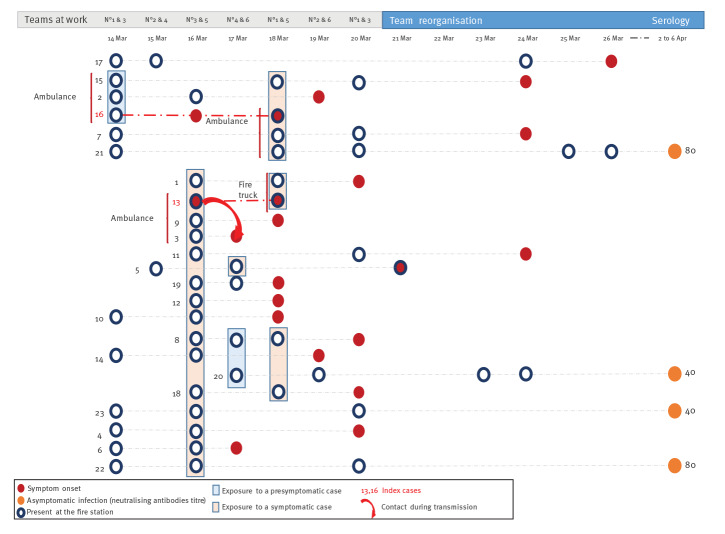
Transmission chains, COVID-19 outbreak at a fire station, Marseille, France, 2020 (n = 23 cases)

Given the history of illnesses, the team organisation and the low-level circulation of the virus in this city at this time, we propose two transmission chains from these index cases (Figure 4). The first index case (Case 16) worked in an ambulance 2 days before onset (14 March, Teams 1 and 3) with two other firefighters who became sick 2 and 5 days later. Two days after onset (18 March), he also worked in an ambulance with two other firefighters: one became sick 6 days later, and the other was found to have neutralising antibodies without declaring any symptoms. The second index case (Case 13) became sick on 16 March while he was working. Of the 16 firefighters present that day (Teams 3 and 5), 13 became sick in the next few days (mean: 3.1 days, range: 1–8 days) and two developed neutralising antibodies. The latter two and four other sick firefighters were also present on 14 March during the presymptomatic period of the first index case. During this outbreak, team work was reorganised, and Teams 1, 3 and 5 and Teams 2, 4, and 6 worked together. We found an attack rate of eight in 14 for Team 3, seven in 14 for Team 5, four in 14 for Team 1 and one in 13 for the other teams.

COVID-19: coronavirus disease.

Only the confirmed cases and their periods of activity are presented in the figure. From 21 March onwards, the working rule was a maximum of 48 h consecutive activity followed by at least 48 h of rest. Transmission refers to the overlaps time between two teams.

## Workplace safety plan and outbreak control measures

Before the outbreak, the 82 operational firefighters were split into six teams of 13–14 men, numbered Team 1–6. The remaining three persons present at the fire station were administrative and management personnel. Operational teams worked together in pairs for 24 h — Teams 1 and 3, Teams 2 and 4, Teams 3 and 5, and Teams 4 and 6 — and then rested for 24 h. We conducted interviews with the fire station’s management to identify key points of the workplace safety plan implemented after the first cases were diagnosed (21 March) to contain the spread of the virus. The workplace safety plan was based on three key points. Firstly, symptoms were closely monitored for early detection of possible cases. Presence of symptoms led to systematic molecular diagnosis and isolation. Firefighters who had any symptoms were not allowed to come to work. Nevertheless, no replacement staff was brought in during that period. Symptomatic staff had to stay at home after the onset of symptoms, even if mild, until 2 days after the symptoms ended, whatever the result of their RT-PCR test. Secondly, social distancing, hand washing and general hygiene measures were implemented. Mask wearing was not mandatory, even in a vehicle, except during medical rescue interventions where all safety measures were taken and personal protective equipment was worn (gown, gloves, mask, glasses). Thirdly, the station’s organisation was modified on 21 March to create two operational groups that alternated and never crossed paths (Figure 2). Each group worked a maximum of 48 h and then rested at least 48 h. As observed in the epidemic curve, the incidence decreased after this reorganisation (Figures 2 and 4).

## Discussion

Here we describe an outbreak of COVID-19 at a fire station. This context provided an opportunity to track virus transmission, since firefighters live in close contact with one another, sharing the same facilities and vehicles day and night. At the time of this outbreak, SARS-CoV-2 was circulating at a low level in the city, and introduction of the virus by a source other than the two index cases is unlikely. Interestingly, index Case 16 illustrates two different kinds of transmission. Firstly, at least two secondary cases (Case 2 and Case 17), and possibly three others, were contaminated during his presymptomatic stage. Case 2 and the index case shared the same vehicle 2 days before. Secondly, the index case developed mild symptoms (asthenia, cough and diarrhoea) that initially were not reported to the station’s medical facility, which explains why he was not isolated. Symptoms and their date of onset were obtained through extensive questioning during the investigation stage. After onset, at least two cases could be related to Case 16, highlighting transmission during the paucisymptomatic stage. These two contamination modes, presymptomatic and paucisymptomatic stages, have been previously described [3]. At the onset of the pandemic, it has been proposed that 23% of cases in the city of Shenzen were related to presymptomatic cases, and this percentage increased to 46% after generalisation of an isolation policy [17]. A systematic review found that the transmission from presymptomatic people was higher than from asymptomatic persons. The authors found a risk ratio of 0.35 (95% confidence interval (CI): 0.1–1.27) for asymptomatic compared with symptomatic, and a risk ratio of 0.63 (95% CI: 0.18–2.26) for presymptomatic compared with symptomatic people [18]. Previous modelling of presymptomatic infectiousness estimated that 37–48% of secondary cases occurred during the presymptomatic stage [19]. This is consistent with the preventive public health strategy deployed worldwide consisting of physical distancing, hand hygiene and mask wearing for all, and not only for symptomatic cases. However, we believe that it is important not to confuse asymptomatic (which will never develop symptoms) and presymptomatic cases. Indeed, the four truly asymptomatic cases (Cases 20–23) were not the source of a third generation of cases (despite the fact that they continued working without wearing a mask), clearly indicating that asymptomatic cases did not contribute substantially to this outbreak.

The investigation of this outbreak highlights different specific contexts that played a role in the transmission chain. For instance, Index Case 13 spread the virus during his team’s shift to Case 5 who fell ill 4 days later (and hadn’t returned to work in the meantime). This case appeared to be the source of a superspreading event, since 16 other cases could be linked to him. Index Case 16 spread the virus during team work on the same ambulance vehicle as two other crew members. These two modes of transmission – from presymptomatic and paucisymptomatic carriers – appear to constitute a major risk and should lead to the implementation of strong preventive measures whenever possible. It was not possible in this investigation to evaluate the contamination risk related to shared meals and dormitory accommodations in this community. Finally, we considered the risk of transmission via medical rescue interventions to be low, since firefighters wore personal protective equipment and were familiar with safety procedures.

Despite 19 symptomatic cases, management was effective, with the epidemic being brought under control in 10 days and without mask wearing. Only one second generation of cases occurred. The attack rate was of 27%, lower than those observed in family clusters (up to 100%), or during a COVID-19 outbreak in a French aircraft carrier (67.9%) [20,21]. Reorganising firefighters in two separate teams that never crossed paths was an effective solution to limit transmission without interrupting activities, as demonstrated by the epidemic curve. The strategy based on early detection of clinical signs combined with self-isolation of all symptomatic cases was also effective. All the cases with positive SARS-CoV-2 RT-PCR results at the time of investigation had already been identified and isolated. Only one of them did not seroconvert. Our data suggest that careful screening for symptoms and clinical examination could be more useful in clinical practice than RT-PCR for isolation decision-making. This self-isolation strategy also raises a question about household transmission. Indeed, the isolation of cases at their home protected the population at the fire station but two firefighters reported secondary cases among their family members.

The laboratory investigation allowed us to highlight three interesting findings. Firstly, the early appearance of neutralising antibodies: less than 10 days after onset for four cases, between 11 and 15 days after onset for nine cases and more than 16 days for five cases. This is consistent with Okba et al., who found seroconversion within 2 weeks after disease onset [14]. All except one confirmed SARS-CoV-2-infected case developed measurable and specific immunity. Only one case never developed measurable neutralising antibodies. The second important finding was the 6% seroprevalence in asymptomatic cases. None of the 66 contacts had a positive nasal swab for the virus. This finding is also consistent with the literature, since Long et al. found 6.1% asymptomatic cases in hospitals in China [22]. Choe et al. found neutralising antibodies in seven of seven completely asymptomatic patients previously confirmed by RT-PCR [23]. Interestingly, when compared with symptomatic cases, that study found a GMT correlated with the severity of the illness: a GMT of 78 in the asymptomatic group, a GMT of 256 for subtle pneumonia and a GMT of 3,158 for apparent pneumonia. In our cohort, although none of our symptomatic patients were hospitalised, GMT were comparable between asymptomatic (GMT = 50) and symptomatic (GMT = 64) cases. Finally, we found that five patients were still SARS-CoV-2 RT-PCR-positive between 1 and 9 days after the end of symptoms. This raises the question whether these people are still contagious, even though we were able to cultivate the virus from one sample of a patient who still had symptoms when sampled 13 days after onset.

## Conclusion

This investigation of a COVID-19 cluster among firefighters during the early phase of the COVID-19 epidemic in France, highlights the efficacy of close clinical monitoring, including the consideration of mild symptoms, to enable early isolation of potential cases in order to control the spread of the epidemic. Our investigation showed that transmission from presymptomatic individuals exists and should be prevented using public health strategies. Transmission from asymptomatic cases was not demonstrated during this outbreak. Measurable immunity was found in all confirmed cases.
